# Mortality and Associated Risk Factors in Patients With Candida auris: Insights From a Tertiary Care Hospital in Bahrain

**DOI:** 10.7759/cureus.69699

**Published:** 2024-09-19

**Authors:** Rawan Alagha, Abdulrahman Sharaf, Hanan Abdulkhaleq, Rommel Acunin, Nermin Saeed

**Affiliations:** 1 Infectious Diseases, Government Hospitals, Manama, BHR; 2 Clinical Pharmacy, Government Hospitals, Manama, BHR; 3 Internal Medicine, Government Hospitals, Manama, BHR; 4 Infection Prevention and Control, Government Hospitals, Manama, BHR; 5 Pathology, Government Hospitals, Manama, BHR

**Keywords:** candida auris, colonization, indwelling device, infection control, mortality, risk factors

## Abstract

Introduction

Candida auris poses a significant healthcare challenge due to its high mortality rates, difficulties in identification, and resistance to antifungal treatment. This study aims to identify risk factors associated with 30-day mortality in patients with either invasive Candida auris infection or colonization.

Methods

We conducted a prospective study at Salmaniya Medical Complex, a tertiary care hospital in Bahrain, from September 2023 to February 2024. The study included 59 patients infected or colonized by Candida auris. Data were systematically collected from patient records, including demographics, clinical characteristics, and outcomes. Risk factors for 30-day mortality were analyzed using both univariate and multivariate statistical methods.

Results

Among the 59 patients studied, the mean age was 63.9 years, and the cohort was predominantly male (74.6%). Key findings include a high prevalence of multiple indwelling catheters (44.2%) and recent intubation (42.4%). Candida auris was most frequently isolated from the groin (33.9%) and urine (25.4%), with a notable presence in the axillary regions (23.7%). The mortality rate was 44.1%. Univariate and multivariate analyses revealed that age (≥65 years), multiple indwelling catheters, ICU admission for over 24 hours, and recent intubation were significant risk factors for mortality while chronic kidney disease did not retain its significance in the multivariate model.

Conclusion

The study underscores the critical need for focused infection control and strong antimicrobial stewardship to address the high mortality associated with Candida auris infections. Emphasizing the importance of early detection and a multidisciplinary approach, these strategies are essential for managing and mitigating the impact of Candida auris in healthcare settings.

## Introduction

Candida auris (C. auris), a yeast resistant to multiple antifungal agents, has become a significant burden on healthcare facilities worldwide due to its ability to cause difficult-to-control outbreaks. Its capability to cause invasive infections, coupled with challenges in eradication, stems from its intrinsic ability to form biofilms that resist vigorous elimination efforts and surface decontamination [1]. Recently, the previous sporadically reported cases of C. auris have dramatically changed to reporting hospital outbreaks among those susceptible to creating clusters of infection or colonization [2]. This increase in incidence is attributed not only to host factors but also to the misuse of antimicrobials, including antifungals, and the subsequent pressure exerted on humans and the environment [3].

Risk factors for acquiring C. auris are similar to those associated with other Candida species, including prolonged ICU admission, uncontrolled diabetes mellitus, chronic liver or kidney disease, prior use of antibiotics and antifungals, the presence of urinary or central venous catheters, and extremes of age [4]. Understanding the impact of these risk factors on mortality is crucial for effectively managing and controlling this emerging pathogen. By identifying high-risk individuals, healthcare providers can implement targeted interventions, potentially reducing mortality rates. Additionally, analyzing these risk factors can develop public health strategies, including resource allocation and outbreak prevention measures [5].

C. auris can colonize areas like the groin, nostril, axilla, and urinary and respiratory systems [2,6]. Colonized patients may shed the fungus into the surrounding environment, with the pathogen persisting on reusable medical equipment, such as blood pressure cuffs and thermometers, leading to the potential for horizontal transmission to other patients [7]. Identical genetic patterns have been observed in hospital clusters, indicating transmission within healthcare settings [8,9]. Patients who are colonized are at risk of developing invasive fungal infections, such as candidemia, as demonstrated in previous studies [10]. The mortality rate associated with C. auris outbreaks, as reported in Kuwait, Oman, Russia, Mexico, Saudi Arabia, and Spain between 2016 and 2020, ranges from 17% to 55% but can reach up to 70% [1]. This high morbidity and mortality rate is primarily due to the pathogen’s clade-specific intrinsic resistance to most azoles, particularly fluconazole, as well as its evolving resistance to other agents such as echinocandins and amphotericin B, thereby limiting treatment options [6].

In Bahrain, a Candida auris outbreak emerged at the end of 2021, with clusters identified in the main tertiary care hospital. Patients were either colonized or had invasive infections. Prior to this outbreak, no studies had been published reflecting data from this center, including the risk factors and their impact on hospital mortality rates. The aim of this research is to analyze the risk factors for patients with either invasive Candida auris infection or colonization over a six-month period and to assess these risk factors associated with 30-day mortality.

## Materials and methods

Study design and setting

This prospective chart review study was conducted at Salmaniya Medical Complex, a major tertiary care hospital in the Kingdom of Bahrain, with an approximate capacity of 1,200 beds. The study was conducted over six months, from September 2023 to February 2024, and evaluated all newly identified cases of Candida auris.

Patient population

All patients aged 16 years and older who tested positive for Candida auris during the study period were included, irrespective of the sample type (e.g., blood, urine, respiratory secretions). The study encompassed both infection and colonization cases.

Data collection

Data were systematically collected from patient medical records. The variables documented included demographic information, the site of Candida auris isolation, underlying comorbidities (e.g., diabetes mellitus, hypertension), ICU admission status, prior use of antibiotics and antifungals, presence of indwelling catheters (e.g., central venous, urinary), presence of nasogastric tubes (NGT), and patient outcomes, including mortality. Collected data were tabulated and cleaned in Microsoft Excel (Microsoft Corporation, Redmond, WA, US). Missing data were excluded from the final data for analysis. All patients' personal information was removed from the data to maintain patient information confidentiality.

Study outcomes

The primary objective of this study was to analyze risk factors in patients with invasive Candida auris infection or colonization over a six-month period and assess their association with 30-day mortality.

Ethics statements

Approval has been obtained from the Research Ethics Committee at Governmental Hospitals in Bahrain (Research approval serial no.: 870-108-23; approved on August 01, 2023).

Microbiological identification

Clinical and screening samples with suspected fungal infection were sub-cultured on Sabroud dextrose agar and incubated aerobically at 35 °C. The fungal species were identified using matrix-assisted laser desorption/ionization-time of flight mass spectrometry (MALDI-TOF-MS (Matrix-assisted laser desorption ionization-time of flight mass spectrometry, Bruker Corporation, Billerica, MA, US).

Statistical analysis

The mean and standard deviations were used for the descriptive analysis of metric variables while frequency and proportion (%) were given for categorical variables. Univariate and multivariate analyses were performed to determine the prognostic factor of mortality. Values were considered significant with a p-value of less than 0.05. All analyses were performed using the software program Statistical Packages for Software Sciences (SPSS) version 26 (IBM Corp., Armonk, NY, US).

## Results

A total of 59 patients were included in the study, with a mean age of 63.9 years (SD ± 19.3), almost equally distributed between those under 65 years (49.2%, n=29) and those 65 years or older (50.8%, n=30). The cohort was predominantly male (74.6%, n=44) while females accounted for 25.4% (n=15). Indwelling catheters were common, with 44.2% (n=23) having multiple types of catheters. Catheter use included bladder drainage (44.2%, n=23), central lines (3.8%, n=2), percutaneous endoscopic gastrostomy (PEG) tubes (3.8%, n=2), chest tubes (1.9%, n=1), and nasogastric tubes (1.9%, n=1). Candida auris was most frequently isolated from the groin (33.9%, n=20) and urine (25.4%, n=15), with notable occurrences in the axillary region (23.7%, n=14). Other isolation sites included blood (10.2%, n=6), deep tracheal aspirate (5.1%, n=3), and other fluids (1.7%, n=1). Clinically, 93.2% (n=55) of patients had used antibiotics in the last 90 days, and 22.0% (n=13) had received antifungal treatment. Additionally, 42.4% (n=25) had been intubated, and 22.0% (n=13) had stayed in the ICU for over 24 hours within the last 90 days. Comorbidities were prevalent, with 52.5% (n=31) having type 2 diabetes (mean glycated hemoglobin (HbA1c) 57.4 ± 34.9), 67.8% (n=40) diagnosed with hypertension, and 23.7% (n=14) with chronic kidney disease. Cardiovascular disease was observed in 37.3% (n=22), and 6.8% (n=4) were immunocompromised. The study's mortality rate was 44.1% (n=26), with 55.9% (n=33) of patients surviving (Table 1).

**Table 1:** Patient demographic and clinical characteristics (n=59) Data are presented as n (%) for categorical variables and mean ± standard deviation (SD) for continuous variables. CBD, continuous bladder drainage; PEG tube, percutaneous endoscopic gastrostomy tube; NGT, nasogastric tube; DTA, deep tracheal aspiration; CKD, chronic kidney disease; CVD, cardiovascular disease

Study variables	N (%)
Age in years (mean ± SD)	63.9 ± 19.3
<65 years	29 (49.2%)
≥65 years	30 (50.8%)
Gender	
Male	44 (74.6%)
Female	15 (25.4%)
Indwelling catheter	
CBD	23 (44.2%)
Central line	02 (03.8%)
PEG tube	02 (03.8%)
Chest tube	01 (01.9%)
NGT	01 (01.9%)
Multiple catheters	23 (44.2%)
Site of Candida isolation	
Groin	20 (33.9%)
Urine	15 (25.4%)
Axillary	14 (23.7%)
Blood	06 (10.2%)
DTA	03 (05.1%)
Other fluids/Site	01 (01.7%)
ICU admission more than 24 hours in the last 90 days	13 (22.0%)
Intubation in the last 90 days	25 (42.4%)
Antibiotics in the last 90 days	55 (93.2%)
Antifungal in the last 90 days	13 (22.0%)
Type 2 diabetes	31 (52.5%)
HbA1c (mean ± SD)	57.4 ± 34.9
Hypertension	40 (67.8%)
CKD	14 (23.7%)
CVD	22 (37.3%)
Immunocompromised	04 (06.8%)
Outcome	
Dead	26 (44.1%)
Alive	33 (55.9%)

Risk factors for 30-day mortality in Candida auris: univariate and multivariate analysis

**Figure 1:**
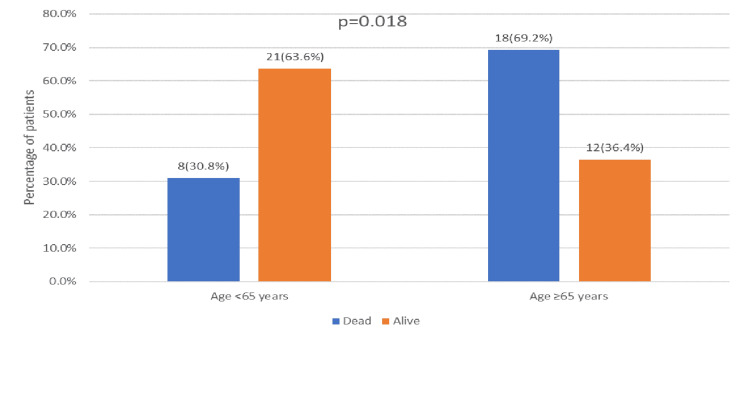
Age group distribution in relation to outcome

**Figure 2:**
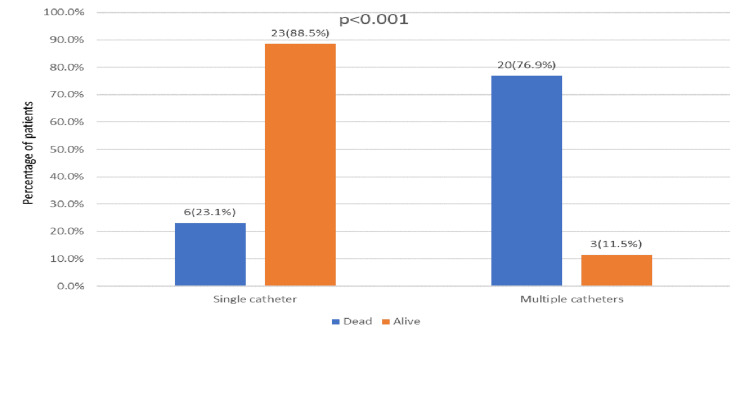
Indwelling catheter distribution in relation to outcome

**Figure 3:**
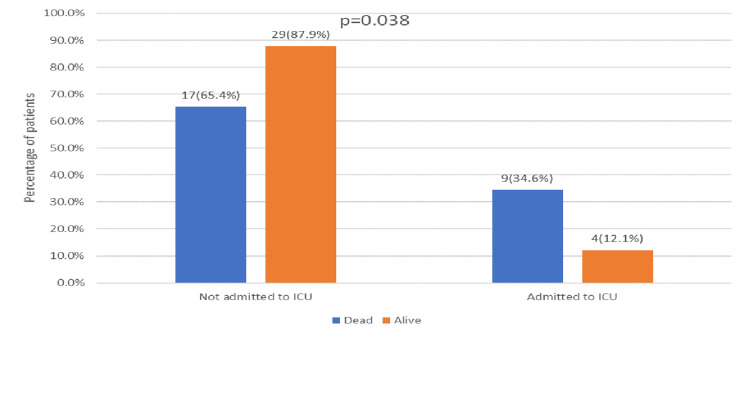
ICU admission >24 hours in the last 90 days in relation to outcome

**Figure 4:**
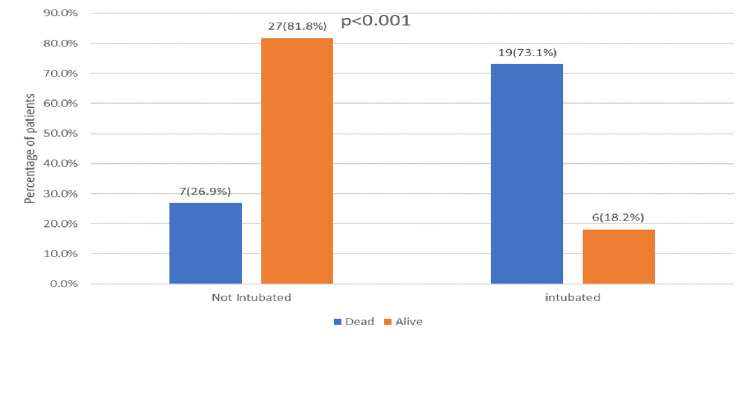
Intubation in the last 90 days in relation to outcome

In the univariate analysis, several risk factors showed significant associations with 30-day mortality in patients with Candida auris. Mortality was notably higher in patients aged 65 years or older, with 69.2% (n=18) of those ≥65 years dying compared to 30.8% (n=8) of those <65 years (p = 0.018) (Figure 1). The presence of multiple indwelling catheters was also strongly linked to increased mortality, with 76.9% (n=20) of those with multiple catheters dying compared to 23.1% (n=6) with single catheters (p < 0.001) (Figure 2). Additionally, ICU admission for more than 24 hours in the last 90 days was associated with higher mortality, with 34.6% (n=9) of these patients dying compared to 12.1% (n=4) who were not admitted to the ICU (p = 0.038) (Figure 3). Intubation within the last 90 days was significantly associated with mortality, affecting 73.1% (n=19) of intubated patients compared to 18.2% (n=6) of non-intubated patients (p < 0.001) (Figure 4). Chronic kidney disease (CKD) also contributed to higher mortality, with 38.5% (n=10) of CKD patients dying compared to 2.1% (n=4) without CKD (p = 0.030). Other factors, such as gender, site of Candida isolation, recent antibiotic and antifungal use, type 2 diabetes, hypertension, cardiovascular disease, and immunocompromised status, did not significantly impact mortality (p > 0.05) (Table 2).

**Table 2:** Univariate analysis of the factors that influence mortality (n=59) Data are presented as n (%) for categorical variables, mean ± standard deviation (SD) for continuous variables, and p-values indicate the significance of differences or associations. § p-value has been calculated using Fisher's exact test. ‡ P-value has been calculated using the independent sample t-test. ** Significant at the p<0.05 level. ICU, intensive care unit; CKD, chronic kidney disease; CVD, cardiovascular disease; NGT, nasogastric tube

Factor	Outcome		P-value ^§^
	Dead N (%) (n=26)	Alive N (%) (n=33)	
Age group			
<65 years	08 (30.8%)	21 (63.6%)	0.018 **
≥65 years	18 (69.2%)	12 (36.4%)	
Gender			
Male	17 (65.4%)	27 (81.8%)	0.229
Female	09 (34.6%)	06 (18.2%)	
Indwelling catheter			
Single catheter	06 (23.1%)	23 (88.5%)	<0.001 **
Multiple catheters	20 (76.9%)	03 (11.5%)	
Site of Candida isolation			
Axillary	06 (23.1%)	08 (24.2%)	0.231
Groin	06 (23.1%)	14 (42.4%)	
Urine	07 (26.9%)	08 (24.2%)	
Other fluids/Site	07 (26.9%)	03 (09.1%)	
ICU admission >24 hours in the last 90 days	09 (34.6%)	04 (12.1%)	0.038 **
Intubation in the last 90 days	19 (73.1%)	06 (18.2%)	<0.001 **
Antibiotics in the last 90 days	25 (96.2%)	30 (90.9%)	0.623
Antifungal in the last 90 days	07 (26.9%)	06 (18.2%)	0.531
Type 2 diabetes	16 (61.5%)	15 (45.5%)	0.295
HbA1c (mean ± SD)	57.1 ± 36.3	57.5 ± 35.5	0.979 ^‡^
Hypertension	19 (73.1%)	21 (63.6%)	0.577
CKD	10 (38.5%)	04 (2.1%)	0.030 **
CVD	13 (50.0%)	09 (27.3%)	0.105
Immunocompromised	02 (07.7%)	02 (06.1%)	1.000

The multivariate analysis, adjusted for potential confounders, confirmed the significance of several risk factors for 30-day mortality. Age ≥65 years remained a significant predictor of mortality with an adjusted odds ratio (AOR) of 4.267 (95% CI 1.194 - 15.248, p = 0.026). The presence of multiple indwelling catheters was a strong predictor of mortality, with an AOR of 29.529 (95% CI 5.522 - 157.898, p < 0.001). ICU admission for more than 24 hours in the last 90 days was also significant, with an AOR of 7.749 (95% CI 1.432 - 41.925, p = 0.017). Recent intubation showed a high association with mortality, with an AOR of 29.076 (95% CI 4.921 - 171.810, p < 0.001). CKD did not retain significance in the multivariate model, with an AOR of 4.031 (95% CI 0.828 - 19.632, p = 0.084) (Table 3).

**Table 3:** Multivariate regression analysis to determine the prognostic factors of mortality (n=59) Adjusted odds ratios (AORs) are reported with 95% confidence intervals (CI), and p-values indicate the significance of differences or associations. Adjusted with gender, diabetes, hypertension, and cardiovascular disease Significant at the p<0.05 level ICU, intensive care unit; CKD, chronic kidney disease; NGT, nasogastric tube

Factor	AOR	95% CI	P-value
Age group			
<65 years	Ref		
≥65 years	4.267	1.194 – 15.248	0.026 **
Indwelling catheter			
Single catheter	Ref		
Multiple catheters	29.529	5.522 – 157.898	<0.001 **
ICU admission >24 hours in the last 90 days			
No	Ref		
Yes	7.749	1.432 – 41.925	0.017 **
Intubation in the last 90 days			
No	Ref		
Yes	29.076	4.921 – 171.810	<0.001 **
CKD			
No	Ref		
Yes	4.031	0.828 – 19.632	0.084

## Discussion

This study identified key risk factors associated with 30-day mortality in patients with Candida auris infections or colonization. Among the 59 patients evaluated, advanced age (≥65 years), multiple indwelling catheters, prolonged ICU admission, and recent intubation were significantly linked to increased mortality. Multivariate analysis confirmed these factors as independent predictors of mortality, highlighting the critical need for focused clinical management in high-risk patients to improve outcomes.

The mortality rate in our study was approximately 44%, with the majority of cases occurring in patients aged 65 years or older. This age group is inherently more vulnerable and typically experiences prolonged hospital stays, contributing to their higher mortality risk. These findings are consistent with a retrospective study by Reham et al. (2023), which also identified higher mortality among older patients with Candida auris [11]. Candidemia can have a mortality rate of up to 40%; however, with C. auris, it can be as high as 70% [12]. High mortality rates were reported in Panama (78%) and India (50%), and data from the Center for Disease Control (CDC) showed that C. auris bloodstream infection had a 39% and 58% mortality rate in 30-day and 90-day observation, respectively [13].

In our study, prolonged hospital and ICU admissions were identified as significant risk factors for mortality, with a rate of 34.6%. This finding aligns with the results of a retrospective study conducted by Muneeba et al.(2019), which reported a clinically significant association between prolonged ICU stays and mortality, with a rate of 46.7% [14].

We observed a strong association between the presence of multiple indwelling catheters and mortality, with a mortality rate of 76.9%. This underscores the challenges in managing patients with multiple devices and the potential for increased biofilm formation and prolonged hospitalization. This finding is consistent with a retrospective study by Hala et al. (2023), which demonstrated that invasive devices are significantly associated with higher mortality among Candida auris patients [15].

In our study, we noticed a predominance of male patients over the six-month observation period. This finding aligns with the results of a meta-analysis by John et al., which analyzed data from 16 countries experiencing Candida auris outbreaks and found a male incidence rate of 64.76%. The reason for this gender distribution remains unclear [12].

Since its discovery in Japan in 2009, Candida auris has emerged as a significant healthcare threat, known for its high mortality rates, challenges in proper microbiological identification, drug resistance, and the risk of persistent infections [12]. The first outbreak in our hospital occurred between November and December 2021, necessitating a coordinated effort among healthcare workers, particularly the infection control unit, microbiologists, and infectious disease specialists. The primary focus was on understanding the severity of the situation, controlling its spread despite the limited availability of isolation rooms and cubicles, and educating nurses and physicians on the importance of strict adherence to contact precautions. Treatment posed an additional challenge, with the emergence of echinocandin resistance in some cases compounding the pre-existing resistance to azoles.

In our hospital, the outbreak occurred at the end of the COVID-19 pandemic, which could have indirectly played a role in its emergence. The C. auris outbreak occurred in almost all countries with variable reported cases in Europe, including Italy, Spain, and the United Kingdom during the COVID-19 pandemic. During that time, healthcare systems were overloaded with the pandemic and were unable to maintain proper stewardship programs running as usual. In addition, infection control measures were overwhelmed [16,17].

Once a Candida auris case is identified, robust measures must be implemented to prevent its spread and avoid a hospital outbreak. The high rate of antibiotic and antifungal use prior to the isolation of C. auris serves as a warning, emphasizing the need for stricter use of these agents and reinforcing the importance of stewardship programs to reduce the incidence of not only C. auris but also other multi-drug resistant organisms. Identifying risks is crucial, and a multidisciplinary team is essential to enforce strict infection control measures. Each case should be thoroughly reviewed, all contacts identified and screened, and patients isolated in single rooms or cohorts. Additionally, surface cleaning with agents that have documented fungicidal activity is necessary to minimize patient-to-environment and environment-to-patient transmission in healthcare facilities [18].

Study limitations

This study is limited by the lack of antifungal susceptibility testing and details on antifungal use, which may have provided additional insights into treatment outcomes. Additionally, the absence of Kaplan-Meier analysis, a small sample size, and no control group limit the generalizability of our findings. Moreover, the distinction between Candida auris colonization and infection was not clearly made due to the limited number of blood samples, which could have influenced the results. Given these limitations, future research should prioritize larger, controlled studies that assess antifungal susceptibility and differentiate between colonization and infection to better inform clinical management strategies.

## Conclusions

Effective management of Candida auris infections requires understanding key risk factors for 30-day mortality. This study identifies advanced age, multiple indwelling catheters, prolonged ICU stays, and recent intubation as significant contributors. The findings highlight the urgent need for improved infection control practices and stringent antimicrobial stewardship. Emphasizing early detection and multidisciplinary collaboration is crucial for managing outbreaks and mitigating the impact of this emerging pathogen in healthcare settings.
